# Supplementary material for: Evidence supported by Mendelian randomization: impact on inflammatory factors in knee osteoarthritis
Source: Front Med (Lausanne). 2024 May 28;11:1382836. doi: 10.3389/fmed.2024.1382836 (PMC11165061; doi:10.3389/fmed.2024.1382836)
Supplement: Supplementary file 1 [file Data_Sheet_1.ZIP › data/GCST90274774_5e6/774.forest.pdf]

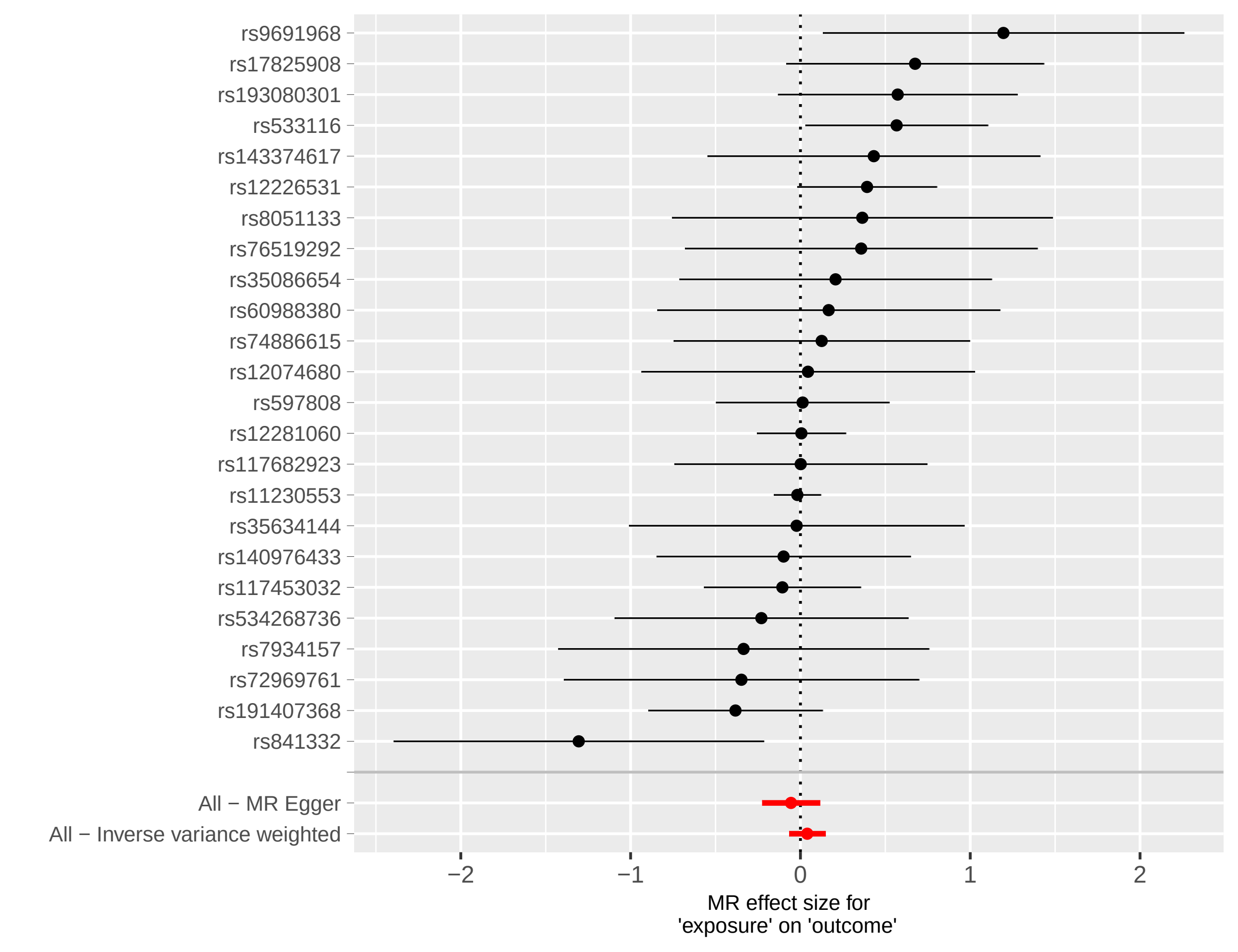

rs9691968
rs17825908
rs193080301
rs533116
rs143374617
rs12226531
rs8051133
rs76519292
rs35086654
rs60988380
rs74886615
rs12074680
rs597808
rs12281060
rs117682923
rs11230553
rs35634144
rs140976433
rs117453032
rs534268736
rs7934157
rs72969761
rs191407368
rs841332
All − MR Egger
All − Inverse variance weighted
−2
−1
0
1
2
MR effect size for
'exposure' on 'outcome'
